# Elasticity of the Achilles Tendon in Individuals With and Without Plantar Fasciitis: A Shear Wave Elastography Study

**DOI:** 10.3389/fphys.2021.686631

**Published:** 2021-06-21

**Authors:** Weiyi Pan, Jiping Zhou, Yuyi Lin, Zhijie Zhang, Yulong Wang

**Affiliations:** ^1^Shenzhen Dapeng New District Nan’ao People’s Hospital, Branch of the First Affiliate of Shenzhen University, Shenzhen, China; ^2^Luoyang Orthopedics Hospital of Henan Province, Luoyang, China; ^3^ShenZhen Second People’s Hospital, The First Affiliated Hospital of Shenzhen University, Shenzhen, China

**Keywords:** elasticity, Achilles tendon, plantar fasciitis, shear wave elastography, rehabilitation

## Abstract

The elastic properties of the Achilles tendon (AT) are altered in local injury or other diseases and in response to changes in mechanical load. Recently, elastography has been used to evaluate variations in tendon elastic properties, mainly among healthy individuals or athletes. Therefore, this study evaluated the biomechanical changes in ATs in individuals with and without plantar fasciitis (PF). The purposes were as follows: (1) to evaluate the passive stiffness of three regions of the AT which defined as 0 (AT0 cm), 3 (AT3 cm), and 6 cm (AT6 cm) above the calcaneal tuberosity in participants with and without PF, (2) to investigate the interplay between the passive stiffness in patients with PF and pain, (3) to detect optimal cut-off points of stiffness of the AT in assessing individuals with chronic PF, and (4) to determine the correlation between the plantar fascia thickness (PFT) and pain. This cross-sectional study included 40 participants (mean age = 51 ± 13 years). When the ankle was in a relaxed position, patients with PF experienced increased passive stiffness in AT0 cm (*p* = 0.006) and AT3 cm (*P* = 0.003), but not in the neutral position. Significant correlations were observed between pain and stiffness of AT (AT0 cm *r* = 0.489, *P* = 0.029; AT3 cm *r* = 487, *P* = 0.030; AT6 cm *r* = 0.471, *P* = 0.036), but not in the PFT (*P* = 0.557). Optimal cut-off stiffness was AT (452 kPa) in the relaxed ankle position. The plantar fascia of patients with PF was significantly thicker than that of the controls (*P* < 0.001). Findings from the present study demonstrate that tendon stiffness is a good indicator of the clinical situation of patients with PF. Monitoring passive tendon stiffness may provide additional information to assess severity of the condition and guide therapeutic. The treatment programs for PF should also be tailored to the distal AT, as conventional therapy might not be targeted to tight tendons.

## Introduction

Plantar fasciitis (PF) is a common disorder of the foot, with an incidence of approximately 1% (Siriphorn and Eksakulkla, 2020). Although PF pain can be alleviated by conservative management, such as a variety of stretching methods, including the Achilles tendon (AT) and plantar fascia, relapse can easily occur. It is generally accepted that the most common causes of PF include tightness of the AT and/or plantar fascia (Nakale et al., 2018). From the perspective of anatomy, the AT is connected to the plantar fascia via the highly regular aligned calcaneal trabeculae of the posterior calcaneus (Zwirner et al., 2020). These published findings all demonstrate a close functional relationship between the AT and plantar fascia. However, the biomechanical effects of AT on plantar fascia have not yet been fully addressed. A better understanding of the pathological changes in the AT of patients with PF will not only enable rehabilitative physicians to better diagnose ankle joint function, but also assist therapists with formulating more effective and targeted treatment programs.

The AT is one of the strongest tendons in the body. It is the elastic structure that connects the triceps surae muscle to the plantar fascia, serves as the main plantarflexing mechanism of the ankle joint, and transmits force based on the amount of stretch endured (Zwirner et al., 2020). Tendon stiffness is a critical determinant of proper muscle force transmission and movement generation (Matijevich et al., 2018). The plantar fascia serves as the primary arch-supporting structure of the foot and tolerates high tension during standing, running, and jumping (Thierfelder et al., 2018). Previous studies have demonstrated that the increased tension of the AT resulting from intense muscle contraction was coupled with the increased strain on the plantar fascia (Cheung et al., 2006). In addition, laboratory testing of the relationship between tensile force in the AT and plantar fascia strain was conducted using eight cadaver lower extremities and revealed that increased AT tension was associated with increased plantar fascia strain (Carlson et al., 2000). Thus, the excessive force on the AT may result in PF, as it alters the normal biomechanical properties of the ankle. However, musculoskeletal simulations or autopsy observations have extensive application for clinical investigations and biomechanical research, and the accuracy of these observations may not be sensitive enough to provide direct quantification of tendon tension, compared to direct measurements of individuals. Therefore, musculoskeletal simulations or autopsy observations cannot accurately reflect biomechanical changes of the tendon, especially its elastic properties. Therefore, reliable and effective imaging techniques are critical for the quantification and assessment of biomechanical changes in patients with PF.

Shear wave elastography (SWE) is an ultrasonography elastography technology known for fast, accurate, and non-invasive evaluation of tissue stiffness (Zhou et al., 2019a,b; Liu et al., 2020). It generates shear waves through an acoustic radiation force impulse, which propagates through the surrounding tissue and traces the wave back to provide biomechanical information about the tissue of the measured object (Zhou et al., 2019a). It has been widely used for muscles, ligaments, and tendons (Zhou et al., 2019a,b; Liu et al., 2020). Our previous studies also demonstrate that SWE is a reliable and valid imaging technique to assess the elastic properties of tendons and muscle (Zhou et al., 2019b). As described, SWE provides an opportunity to evaluate varieties in the AT and plantar fascia thickness during passive ankle stretching in individuals with PF (Zhou et al., 2019a,b; Liu et al., 2020). Additionally, the AT is particularly amenable to SWE assessment given its relatively superficial anatomic positioning. Although alterations in the elastic properties and thickness have been well demonstrated in healthy individuals or individuals with AT pathology, data regarding the biomechanical changes in measurements with SWE in individuals with PF are more limited. To the best of our knowledge, the biomechanical effects of the AT on the plantar fascia have not been fully addressed. As of yet, no study has examined the biomechanical properties of tendon tension in individuals with and without PF, and the relationship between tension and pain.

Therefore, we used Young’s modulus measurements as an index of tendon elastic properties. The purposes of this study were fourfold: (1) to evaluate the passive stiffness of different regions of the AT in participants with and without PF, (2) to investigate the interplay between the passive stiffness in patients with PF and pain, (3) to detect optimal cut-off points of stiffness of the AT to assess chronic PF, and (4) to determine the correlation between the plantar fascia thickness (PFT) and pain.

## Materials and Methods

### Ethics Statement

The study was approved by the Human Subject Ethics Committee of the Luoyang Orthopaedic Hospital of Henan Province (NO: KY2019-001-01). The experimental procedures were explained to each participant prior to participation. All participants provided written informed consent prior to the experiment, and all study procedures adhered to the principles of the Declaration of Helsinki.

### Participants

This study comprised of 40 participants, 20 patients with PF and 20 asymptomatic, aged 32–69 years (mean age 51 ± 13 years for asymptomatic participants and mean age 51 ± 13 years for patients with PF. There were 10 males and 10 females in each group). The inclusion criteria for PF were as follows: (1) plantar heel tenderness, (2) pain symptoms longer than 6 months, (3) morning pain with the first few steps out of bed, (4) pain score ≥ 3/10 using a visual analog scale (VAS), and (5) the more painful leg of patients with bilateral PF was included (BlaBolgla et al., 2015). The exclusion criteria were as follows: (1) neuromuscular disease, musculoskeletal injury of the lower limb, tendon rupture, or anomaly on ultrasound; (2) any previous foot or ankle pathology, such as infections, fracture, or surgery; (3) received any treatment about for the foot or ankle; and (4) need for chronic analgesics due to another condition. The inclusion criteria of 20 age-matched and sex-matched asymptomatic participants were as follows: healthy, no plantar heel tenderness, and meet the exclusion criteria of PF group.

### Demography Characteristics

Basic information about the participants was recorded, including age, sex, height, weight, BMI, VAS score, and duration of pain.

### Equipment and Parameter Setting

The Young’s modulus of the AT was quantified using an ultrasound shear wave elastography system (SuperSonic Imagine, Aix-en Provence, France) with a 40 mm linear array transducer (2–10 MHz, SL10-2). The settings were set as follows (Zhou et al., 2019a,b; Liu et al., 2020): the initial condition was standard musculoskeletal mode. The opacity was 85%. The elastic modulus of the AT was 0–800 kPa, and the preset of B-scan ultrasound was adjusted to a depth of 0–2.5 cm. In the SWE examination, the Q-box diameter of AT was defined as the thickness of the AT (Liu et al., 2020). The color scale used in the Young’s modulus (in kPa) showed the lowest values in blue and the highest values in red.

### Examination Procedures

Participants were placed in a prone position with their feet fully relaxed and placed over the edge of the examination bed. The Young’s modulus of the AT was examined on the painful leg when the ankle was passively “relaxed” or neutrally placed (90°). Leg-matched asymptomatic participants were examined as controls. It is noteworthy that for the relaxed position of the ankle participants took what they perceived to be a “relaxed” ankle position, that is, the feet were maintained in a naturally hanging position, while the neutral (90°) position was fixed by a customized and movable ankle foot orthosis. The angle of ankle of PF group included as the angle of “relaxed” position was tested by a manual goniometer (Sammons Preston, Royan, Canada), and it was used as the relaxation angle of age-matched and gender matched asymptomatic group. As AT has unique twisted structure and anatomical composition, there are some differences in biomechanical characteristics among different positions of AT (Ballal et al., 2014). According to our previous studies, the three regions of AT were defined as 0, 3, and 6 cm above the calcaneal tuberosity (Liu et al., 2020). All corresponding skin surfaces were marked with a black pen. To avoid any effect of tendon fatigue (if any) on the elastic properties, the passive task at the relaxed position was performed before the passive task at the neutral position.

### Achilles Tendon Measurement

**FIGURE 1:**
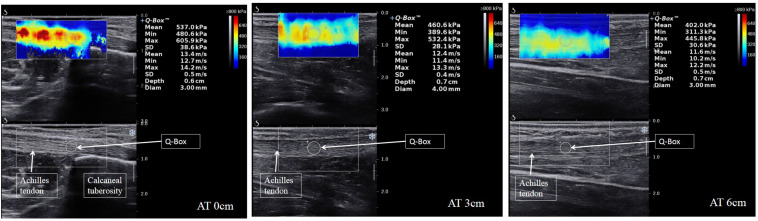
Longitudinal shear wave elastography images of the three regions of the AT in patients with PF show the values of the Young’s modulus at a relaxed ankle position. The Q-Box^TM^ is shown on the right of each scale sonogram.

As proposed previously (Zhou et al., 2019a,b; Liu et al., 2020), a large quantity of ultrasound gel was placed on the AT surface and the transducer was placed at the marker point without applying pressure. Then, activated B-mode ultrasound was adjusted to display the superior-inferior borders of the AT under the longitudinal section. The images were frozen until the images in the region of interest were clear and stable. The Q-box was then placed to calculate the shear modules (Figure 1). This examination procedure was repeated three times in the same region, and the images and values were stored in the equipment.

### Plantar Fascia Thickness Measurement

**FIGURE 2:**
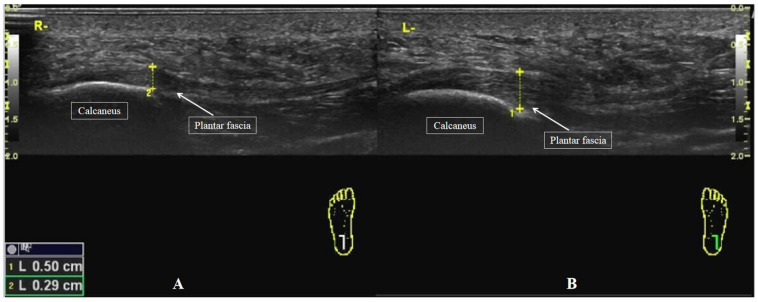
Individual example of a longitudinal sonogram of the PFT. The PFT was assessed at the anterior margin of the calcaneus. **(A)** The PFT of a healthy participant. **(B)** The PFT of a patient with PF.

Participants were placed in the prone position as mentioned above, and a 40 mm linear array transducer (SL10-2, Supersonic Imagine, France) was used to measure the sagittal plantar fascia thickness (PFT). The transducer was placed over the surface closest to the medial foot, and then the PFT was measured at the anterior margin of the calcaneus and stored for offline analyses (Figure 2; Gamba et al., 2018; Gatz et al., 2020; Petrofsky et al., 2020).

### Statistical Analyses

G^∗^Power 3.0.10 software was used to determine the required number of participants. According to the results of a previous pilot study, an estimated effect size of 0.3, was set as 0.05, and the power was 0.85; therefore, the total sample size was 30 participants (i.e., 15 per group). To avoid issues due to dropouts or missing data, the number was increased to 40 (i.e., 20 per group).

The data were analyzed using SPSS version 22.0 software (IBM Corporation, Armonk, NY, United States) and expressed as mean ± standard deviation. The normality of distributions was assessed using the Shapiro-Wilk test. Our data were normally distributed (all *p* > 0.05). Independent *t*-tests were performed for demographic data, such as age, weight, and height. Multivariate analysis of covariance (MANCOVA) tests were performed to compare the Young’s modulus of the ATs (AT0 cm, AT3 cm, and AT6 cm) at different ankle angles (relaxed or neutral) on the painful side of patients with PF and the dominant side of healthy controls, with variables with significant group differences as covariates. When the level of significance was reached, *post hoc* analyses were performed using univariate analysis of covariance tests. Pearson correlation analysis (r) was used to examine the correlation among the main variables (Young’s modulus of ATs), duration of pain and pain, and to examine the correlation between secondary variables (PFT) and pain. Receiver operating characteristic (ROC) curve analysis was performed to calculate the cut-off point of the elastic properties of the ATs that displayed significant group differences. For all tests, statistical significance was set at *p* < 0.05.

## Results

### Demographic Data

Twenty-two participants reported heel pain at the medial plantar and clinical diagnosis suggested the presence of PF, and two candidates declined to participate. Twenty-three asymptomatic participants were assessed for study eligibility, and three did not meet the inclusion criteria. The age, height, weight, and BMI in both groups, VAS score, and duration of heel pain for symptomatic participants are summarized in Table 1. There were no significant differences in age, height, weight, or BMI (*P* > 0.05). However, the PFT of the PF group (0.5 ± 0.8 cm) was significantly thicker than that of the healthy group (0.3 ± 0.1 cm) (by 66.67%; *P* < 0.001). The angle of “relaxed” position of the ankle joint was 23 ± 2 degrees, which was significant difference with the neutral (90°) position (*P* < 0.001).

**TABLE 1:** Demographic data of subjects.

	PF group (*n* = 20)	Healthy group (*n* = 20)	*P*-values
Age (years)	51 ± 13	51 ± 13	0.941
Weight (kg)	66 ± 12	66 ± 11	0.340
Height (cm)	166 ± 8	166 ± 9	0.970
Body Mass Index (kg/m^2^)	25 ± 2	24 ± 3	0.136
Thickness of plantar fascia (cm)	0.5 ± 0.8	0.3 ± 0.1	0.000*
Duration of heel pain (months)	11 ± 5		
VAS score	4 ± 1		

Values are mean ± SD; Healthy subjects: Female 10 (ages 36–69 years), Male10 (ages 33–62 years); Patients with plantar fasciitis: Female 10 (ages 32–69 years), Male10 (ages 35–69 years). * Denotes significant difference between groups (p < 0.05).

### Stiffness of the AT Between PF Group and Healthy Group

The mean elastic property values of the AT in the PF and healthy groups are summarized in Table 2. In the relaxed position, the elastic property values of the AT0 cm and AT3 cm of the PF group were significantly higher than those of the healthy group (13 ± 20%, *P* = 0.006; 10 ± 14%, *p* = 0.003, respectively). There was no significant difference at the AT6 cm. No between-group differences were observed in the three regions of AT measured in the neutral position (*p* > 0.05).

**TABLE 2:** Comparisons of Young’s modulus of the ATs on the painful side of patients with PF and the control subjects.

Variables elastic modulus (kPa)			Healthy group	PF group	Percentage change (%)	*P*-values
Relaxed position	AT	0 cm	445 ± 43	497 ± 66	13 ± 20	0.006*
		3 cm	411 ± 39	449 ± 36	10 ± 14	0.003*
		6 cm	437 ± 39	444 ± 42	3 ± 17	0.565
Neutral position	AT	0 cm	574 ± 57	610 ± 86	7 ± 19	0.133
		3 cm	561 ± 59	601 ± 70	8 ± 17	0.241
		6 cm	568 ± 48	596 ± 42	6 ± 12	0.257

AT, Achilles tendon; AT0 cm, Position of the Achilles tendon at 0 cm above the calcaneal tuberosity; AT3 cm, Position of the Achilles tendon at 3 cm above the calcaneal tuberosity; AT6 cm, Position of the Achilles tendon at 6 cm above the calcaneal tuberosity. *Denotes significant difference between groups (p < 0.05).

### Correlations Among ATs Stiffness, PFT and Pain

**FIGURE 3:**
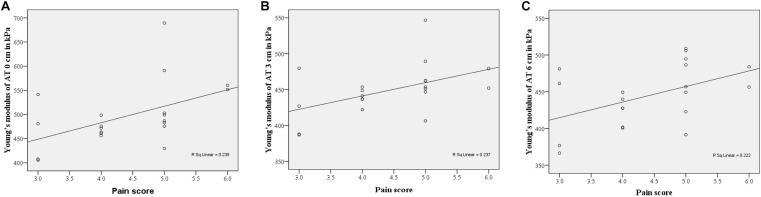
Scatter plots were showed the correlations between the pain score and the elastic properties of ATs at relaxed position. **(A)** AT0 cm, *r* = 0.489, *P* = 0.029, **(B)** AT3 cm, *r* = 0.487, *P* = 0.030, **(C)** AT6 cm, *r* = 0.471, *P* = 0.036.

The correlations among PFT, elastic properties of ATs, and pain scores are showed in Figure 3. When the ankle was in a relaxed position, significant correlations were detected between the pain score and the elastic properties of AT0 cm (*r* = 0.489, *p* = 0.029), the Young’s modulus of AT3 cm (*r* = 0.487, *p* = 0.030), and the Young’s modulus of AT6 cm (*r* = 0.471, *p* = 0.036). There was no significant correlation between pain score and elastic properties of the ATs in the neutral position (*p* > 0.05). In addition, there was no significant correlation between the pain score and PFT (*r* = 0.146, *P* = 0.557).

### Correlations Among ATs Stiffness and the Duration of Heel Pain

The correlations among elastic properties of ATs and the duration of heel pain are summarized in Table 3. There was no significant correlation between ATs stiffness and the duration of heel pain (all *P*-values > 0.05).

**TABLE 3:** Correlations among Young’s modulus of AT and the duration of heel pain.

Variables			*r*	*p*
Relaxation position	AT	0 cm	0.053	0.824
		3 cm	0.007	0.977
		6 cm	0.176	0.459
Neutral position (90°)	AT	0 cm	0.206	0.382
		3 cm	0.059	0.806
		6 cm	0.224	0.342

### Optimal Cut-Off Point in Assessing Chronic PF Patients With SWE

The optimal cut-off point for the elastic properties of ATs for assessing individuals with chronic PF was calculated using ROC curves (Supplementary Figure 1). When considering the ankle in the relaxed position, the AUC of AT0 cm was 0.755 (*p* = 0.006), and that of AT3 cm was 0.755 (*p* = 0.006). Youden’s index revealed that the Young’s modulus of AT (AT0 cm: 452 kPa, sensitivity = 0.85, specificity = 0.40; AT3 cm: 417 kPa, sensitivity = 0.85, specificity = 0.40) at the relaxed position of the ankle in assessing individuals at risk for chronic PF. The AUCs for the rest of the tested regions were not statistically significant and were included in the neutral position.

## Discussion

Taking advantage of SWE, this study evaluated the biomechanical changes in the AT of individuals with and without PF. We observed that the PF group exhibited higher distal AT (AT0 cm and AT3 cm) stiffness in the relaxed position of the ankle. Moreover, significant correlations were detected between pain score and the elastic properties of AT in the relaxed position of the ankle, and no significant correlation between ATs stiffness and the duration of heel pain. Furthermore, the optimal cut-off points of the elastic properties of ATs were determined in the relaxed position of the ankle, which may help to assess individuals with chronic PF.

### Stiffness of the AT Between Individuals With and Without PF

This is the first study to compare the elastic properties of ATs between individuals with PF and healthy controls. As proposed in a recent study, the measurement of passive elastic properties could be advocated as a preventive, diagnostic, and prognostic instrument in individuals with PF. Our results indicate that when the ankle was in a relaxed position, the passive elastic properties of the AT0 cm and AT3 cm of patients with PF were stiffer than those of healthy individuals, but this was not observed for the AT6 cm. Interestingly, there were no significant differences in the passive elastic properties of the ATs between the PF and healthy groups when the ankle was in a neutral position. These findings could be interpreted by the fasciopathy patterns of the histological alterations of PF. In the degenerative processes, matrix degradation, fibroblastic hypertrophy, and collagen breakdown result in a lack of plantar fascia stiffness and contribute to PF (Schillizzi et al., 2020). The stiffer plantar fascia could reduce the length and excitation of the AT, resulting in increased stiffness of the AT (Ostermann et al., 2020). The increased stiffness in the AT could reduce the efficiency of stabilization and plantar flexion of the ankle, and could ultimately compromise the plantar fascia further (Ostermann et al., 2020). Recently, various stretching methods, including both the AT and plantar fascia, have been proven to be effective in mitigating pain in patients with PF (Engkananuwat et al., 2018). From the perspective of biomechanics, increasing strain on the plantar fascia was coupled with increased tension on the AT, and the plantar fascia showed a twofold larger straining effect (Cheung et al., 2006). Reduction of the AT force may decrease the plantar fascia load (Chen et al., 2015). Moreover, previous studies have demonstrated that stretching can decrease tendon stiffness (Zhou et al., 2019a,b; Liu et al., 2020). Therefore, lengthening or tension relief of the AT, especially in participants with tight AT, may be beneficial in terms of plantar fascia stress relief (Cheng et al., 2008). However, the passive elastic properties of ATs were not significantly different between the PF group and the healthy group when the ankle was in a neutral position. These results could be explained by the similar passive tension of AT engendered by the stretching force in individuals with or without PF. Further investigations are needed to verify this, as well as the change in AT stiffness in patients with PF before and after treatment (e.g., extracorporeal shock wave therapy, stretching).

Our results also demonstrated that the percentage change in the AT0 cm (11.50%) stiffness of the participants with PF was significantly higher than that of AT3 cm (9.23%). The disparity in different regions of AT may be due to the different regions of AT experiencing different strains and compressive loads and exhibit distinct histological alterations in PF (Ballal et al., 2014). For example, higher amounts of cartilage cells and cartilage matrix proteins were produced in AT0 cm, which could result in a stiffer tendon structure (Maffulli et al., 2006; Bah et al., 2016), whereas the increase in abnormal tenocytes and type III collagen in AT3 cm could reduce the ability of tendon tissue to resist tensile forces (Ooi et al., 2015). In addition, considering the rotatory anatomy and multiple muscles of the AT, it can be assumed that a difference in AT composition has an impact on the non-uniform behavior of the different regions in the AT (Winnicki et al., 2020). Furthermore, AT0 cm is directly attached to the calcaneus so that it suffers more load-bearing strain than other regions (Zwirner et al., 2020). In conclusion, the stiffness was higher at AT0 cm than at other locations in patients with PF.

### Correlations Among Stiffness, PFT, and Pain

We observed significant correlations between pain and AT stiffness when the ankle was in a relaxed position. This means that the increased the stiffness of the AT is associated with greater intensity of pain. Pearce et al. (2021) found that there was a strong correlation between the severity of heel pain and gastrocnemius tightness in patients with PF (Pearce et al., 2021). The tension of AT is closely related to plantar fascia loading (Schneider et al., 2018). In addition, anatomical studies have shown that a continuation of fibers connects the gastrocnemius via the AT to the plantar fascia (Ballal et al., 2014). Furthermore, previous studies have shown that the success of AT stress relief for treatment of PF, such as stretching. In summary, clinically, reducing the tension or stiffness of AT is very important to improve the outcomes of patients with PF. The stiffness of the AT may play a role in guiding therapeutic interventions in patients with PF. Notably, the degree of plantar fascia thinning in response to a determinate treatment is usually the principal variable evaluated in previous studies but remains somewhat controversial. Mahowald et al. (2011) observed a good correlation between decreased pain and reduced thickness of plantar fascia for 30 patients after receiving various treatments and concluded that changes in the thickness of the plantar fascia are a reliable indicator of the efficacy of treatment protocols for PF. In addition, McMillan et al. (2012) reported a moderate correlation between pain and thickness of the plantar fascia. However, in another study (Maki et al., 2017) the thickness of the plantar fascia did not vary after extracorporeal shock wave therapy (ESWT). On MRI findings before ESWT, the mean thickness of the PF was 4 ± 2 mm, and after ESWT, the thickness was 5 ± 1 mm. Gamba et al. (2018) also found that the PFT in patients with PF should not be used in treatment planning, as the PFT did not correlate with AOFAS, pain, or any item of the SF-36. Therefore, changes in PFT are not a reliable indicator of the efficacy of treatment protocols for PF. The findings of the present study showed that the stiffness of the AT is an important reference parameter in treatment management, as we found a strong correlation between pain and AT stiffness. SWE can provide quantitative data to monitor the efficacy of rehabilitation and treatment protocols. More importantly, this result suggests that releasing AT stiffness is recommended for the treatment of patients with PF. Further studies are needed to establish whether this suggestion is related to clinical benefits.

Our results demonstrate that the plantar fascia is significantly thicker among individuals with PF than healthy participants (0.5 ± 0.8 cm vs. 0.3 ± 0.1 cm), and there was no correlation between PFT and pain, indicating that PFT is not a good clinical indicator of treatment response. The findings of this study are consistent with recent reports. For example, Gatz et al. (2020) reported that the mean PFT of symptomatic participants (0.4 ± 0.1 cm) was thicker than that of asymptomatic participants (0.3 ± 0.1 cm). Petrofsky et al. (2020) also demonstrated a significantly thicker PFT in their PF group compared to controls. In addition, Gamba et al. (2018) observed that the PFT in patients with PF was not associated with its clinical impact, as the PFT was not associated with AOFAS, pain score, or any item of the SF-36. Moreover, although the findings of Alotaibi et al. (2020) indicate that PFT significantly decreased after treatment, no significant correlation was observed between PFT and changes in pain after 4 weeks of treatment. Therefore, PFT could be used as a diagnostic predictor, but not as an indicator of treatment response in PF.

### Correlations Among ATs Stiffness and the Duration of Heel Pain

This study also found that there was no significant correlation between ATs stiffness and the duration of heel pain. Our results are similar to previous studies. Chen et al. (2013) found that there was no significant correlation between symptom duration and pain in patients with plantar fasciitis. This may be related to the self-healing of plantar fascia. Under overload due to multiple causes, the morphological characteristics of the plantar fascia are modified by the process of inflammation (Lareau et al., 2014). In this process, the plantar fascia is swollen and thickened, and pain intensity increases. This could still be reversed by conservative treatment (Fabrikant and Park, 2011; Luffy et al., 2018). However, the inflammation becomes chronic with ultrastructural changes in the plantar fascia if there is no response to treatment and the inflammation process will stop and leave a thickened and degenerated fascia. The pain threshold increases during this entire process, resulting in the illusion of pain relief, and the VAS score decreases (Pomares et al., 2011). In addition, the plantar fascia is capable of self-healing, just as the measure of rest is taken for the affected foot to allow time for healing (Luffy et al., 2018). Clinically, the assessment of the severity of PF mainly depends on clinical symptoms, and imaging diagnosis mainly includes MRI and ultrasound. In addition, some validated scales will be used to evaluate the foot and ankle function and activity, such as foot and ankle ability measure and foot function index (Martin et al., 2014). Therefore, the assessment of the severity of PF needs to combined with multiple examination findings and clinical symptoms.

### Optimal Cut-Off Point in Assessing Chronic PF Patients With SWE

As discussed above, the increase in the passive elastic properties of the AT may be due to an overload of the plantar fascia associated with sports participation. Preventive methods include a variety of stretching methods, including AT, plantar fascia, or silicone insoles. Ultrasound measurements, using both the SWE and grayscale to assess the morphology of the plantar fascia and the AT stiffness, may provide additional information to assess severity of the condition and guide therapeutic interventions in patients with PF. Given this, we calculated the initial cut-off stiffness of the AT (452 kPa, sensitivity = 0.85) at the relaxed position of the ankle to assess chronic PF. This also suggests that individuals with a passive stiffness of the AT greater than 452 kPa may have a higher risk of plantar fascia injury. It is worth noting that the results of ROC curves showed that AUC has relatively low specificity for patients with chronic PF. This may be related to the different activity patterns and exercise intensity between the PF group and healthy group. According to previous studies, the more activity or exercise intensity, the greater the stiffness of AT (Engkananuwat et al., 2018; Schneider et al., 2018; Zhou et al., 2019a,b; Liu et al., 2020; Siriphorn and Eksakulkla, 2020).

### Limitations

The limitations of this study are that electromyography was not used to monitor the gastrocnemius muscle and soleus muscle activity during SWE measures and ensure these muscles were no firing. However, all participants were verbally instructed to stay relaxed, and no signs of muscle contraction were observed on the gray-scale image. Based on this, we are confident that while their lower limb were fully supported, patients or subjects remained in a passive state during passive dorsiflexion. In addition, we did not estimate the correlation between AT stiffness and plantar fascia stiffness in patients with PF. Furthermore, we did not use longitudinal follow-up to observe how AT stiffness and plantar fascia thickness changed during treatment, and whether they were affected by interventions such as stretching. Further studies should be conducted to investigate these factors.

## Conclusion

The present study is the first to assess the relationship between heel pain severity and AT stiffness in patients with PF. Patients with PF had significantly greater AT stiffness than healthy participants. The increase in passive stiffness of distal AT is associated with pain, and initial cut-off points of distal AT stiffness at the ankle relaxed position may provide additional information to assess severity of the condition and guide therapeutic. The treatment programs for PF should also be tailored to the distal AT, as conventional therapy might not be targeted to tight tendons.

## Data Availability Statement

The data analyzed in this study is subject to the following licenses/restrictions: Datasets are available on request. Requests to access these datasets should be directed to reha_jp@163.com.

## Ethics Statement

The studies involving human participants were reviewed and approved by the Human Subject Ethics Committee of the Luoyang Orthopedic Hospital of Henan Province. The patients/participants provided their written informed consent to participate in this study.

## Author Contributions

WP and ZZ designed the experiments. JZ and YL carried out the experiments and collection data. WP and JZ analyzed the experimental results and drafted the manuscript. All authors contributed to manuscript revision, read, and approved the submitted version.

## Conflict of Interest

The authors declare that the research was conducted in the absence of any commercial or financial relationships that could be construed as a potential conflict of interest.

## Supplementary Material

Supplementary Figure 1 The ROC curves for the AT0 cm and AT3 cm in the relaxed position. The AUC of AT0 cm was 0.755 (*P* = 0.006), and that of AT3 cm was 0.755 (*P* = 0.006).
