# Supplementary figures and images for: Elasticity of the Achilles Tendon in Individuals With and Without Plantar Fasciitis: A Shear Wave Elastography Study
Source: Front Physiol. 2021 Jun 21;12:686631. doi: 10.3389/fphys.2021.686631 (PMC8257043; doi:10.3389/fphys.2021.686631)

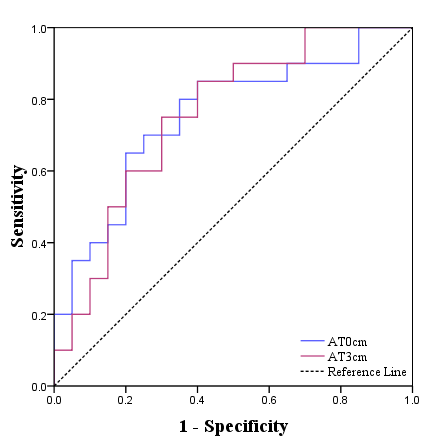

Supplement: Supplementary Figure 1 — The ROC curves for the AT0 cm and AT3 cm in the relaxed position. The AUC of AT0 cm was 0.755 (P = 0.006), and that of AT3 cm was 0.755 (P = 0.006). [file Image_1.PNG]
